# Chromosomal-level genome assembly data from the pale chub, *Zacco platypus* (Jordan & Evermann, 1902)

**DOI:** 10.1016/j.dib.2024.110596

**Published:** 2024-06-13

**Authors:** Sang-Eun Nam, Jae-Sung Rhee

**Affiliations:** a Department of Marine Science, College of Natural Sciences, Incheon National University, Incheon, 22012, South Korea; b Research Institute of Basic Sciences, Incheon National University, Incheon 22012, South Korea; c Yellow Sea Research Institute, Incheon 22012, South Korea

**Keywords:** Xenocyprididae, Fish genome, *Zacco platypus*, *De novo* genome assembly

## Abstract

The pale chub, *Zacco platypus* (Cypriniformes; Xenocyprididae; homotypic synonym: *Opsariichthys platypus*; Jordan & Evermann, 1902), is widely distributed in the freshwater ecosystems throughout East Asia, including South Korea. In this study, we constructed a *de novo* genome assembly of *Z. platypus* to serve as a reference for fundamental and applied research. The assembly was generated using a combination of long-read Pacific Bioscience (PacBio) sequencing, short-read Illumina sequencing, and Hi-C sequencing technologies. The draft genome of *Z. platypus* consisted of 16,422,113 reads from the HiFi library, 702,143,130 reads from the Illumina TruSeq library, and 250,789,660 reads from the Hi-C library. Assembly with Hifiasm resulted in 336 contigs, with an N50 length of 31.9 Mb. The final assembled genome size was 838.6 Mb. Benchmarking Universal Single-Copy Orthologs (BUSCO) analysis indicated that 3,572 (98.1 %) of the expected genes were found in the assembly, with 3,521 (96.7 %) being single-copy and 51 (1.4 %) duplicated after searching against the Actinopterygii database. Of the 319 Hi-C scaffolds, 24 exceeded 10 Mb were thus classified as chromosome-level scaffolds. The assembled genome comprises 41.45 % repeat sequences. Gene annotation was performed using Illumina RNA-Seq and PacBio Iso-Seq data, based on repeat-masked genome sequences. The final annotation resulted in 34,036 protein-coding genes. This chromosomal-level genome assembly is expected to be a valuable resource for future health assessments in aquatic ecosystems, providing insights into the developmental, environmental, and ecological aspects of *Z. platypus*.

Specifications Table

**Table:** 

Subject	Biology
Specific subject area	Genomics
Type of data	Genome assembly, Sequencing raw reads, Table, Figure
Data collection	High molecular weight DNA and RNA were extracted from frozen tissues of Z. platypus. A de novo genome assembly was performed using a combination of long-read PacBio HiFi sequencing, short-read Illumina sequencing, and Hi-C sequencing technologies. Gene annotation was conducted using evidence sequences obtained from Illumina RNA-Seq and PacBio Iso-Seq.
Data source location	37°31′28.0″ N; 127°5′26.0″ E
Data accessibility	Repository name: Whole genome sequencing in *Zacco platypus*Data identification number:Final genome assembly: GCA_038088235GenBank: JBBHEA000000000 (JBBHEA010000001-JBBHEA010000316)BioProject: PRJNA1088288BioSample: SAMN40466320SRA: SRR28903496-SRR28903500Direct URL to data:Final genome assembly: https://www.ncbi.nlm.nih.gov/datasets/genome/GCA_038088235.1/GenBank: https://www.ncbi.nlm.nih.gov/nuccore/JBBHEA000000000.1BioProject: https://www.ncbi.nlm.nih.gov/bioproject/PRJNA1088288BioSample: https://www.ncbi.nlm.nih.gov/biosample/SAMN40466320SRA: • ZP-HiFi-01: https://trace.ncbi.nlm.nih.gov/Traces?run=SRR28903500 • ZP-illumina-DNA-01: https://trace.ncbi.nlm.nih.gov/Traces?run=SRR28903499 • ZP-HiC-01: https://trace.ncbi.nlm.nih.gov/Traces?run=SRR28903498 • ZP-illumina-RNA-01: https://trace.ncbi.nlm.nih.gov/Traces?run=SRR28903496 • ZP-Iso-01: https://trace.ncbi.nlm.nih.gov/Traces?run=SRR28903497
Related research article	None

## 1 Value of the Data


 

• The chromosomal-level genome assembly of *Z. platypus*, constructed using Hi-C assembly, has been made publicly available.
• The high quality of whole-genome sequencing performed in *Z. platypus* was confirmed by the high BUSCO completeness (98.1 %).
• Gene annotation was performed using a *de novo* transcriptome assembly that integrated protein sequences from closely related species, PacBio Iso-Seq, and Illumina RNA-Seq data.
• Because *Z. platypus* is an indicator species for freshwater health assessment, the genome resources provided can offer valuable information to support further studies for health assessment in aquatic ecosystems.

## 2 Background

Freshwater ecosystems globally are threatened by numerous anthropogenic activities, including direct or indirect disposals and runoff from land. Chemical characterization of pollutants in waterbodies can provide partial information for conducting environmental risk assessments. To assess the actual health status of freshwater organisms, aquatic ecotoxicology has been utilizing model organisms such as zebrafish for the risk assessment of pollutants through the application of multi-omics [1,2]. However, these organisms are not indigenous species, and laboratory experiments rarely reflect their natural or real-world settings [3]. Thus, it is difficult to directly apply the results obtained from model animals to the health and risk assessment of domestic aquatic ecosystems. In South Korea, the quality criteria for freshwater are measured using indicator species, which vary according to the specific habitat characteristics [4]. The pale chub, *Zacco platypus* is one of the indicator species used for assessing water quality. However, current water quality criteria are only assessed by the presence or absence of indicator species, rather than through molecular-level analysis. Additionally, there has been less research conducted in the field of multi-omics in these non-model indicator species compared to zebrafish. Therefore, in this study, we constructed a genome database of *Z. platypus* for the health assessment of domestic aquatic ecosystems.

## 3 Data Description

A *de novo* genome assembly of *Z. platypus* was constructed using a combination of long-read Pacific Biosciences (PacBio) platform (Sequel II), short-read Illumina platform (NovaSeq 6000), and Hi-C sequencing technologies (Table 1). The draft genome of *Z. platypus*, containing 16,422,113 reads from the HiFi library, 702,143,130 reads from the Illumina TruSeq library, and 250,789,660 reads from the Hi-C library, was obtained (Table 1). Assembly with Hifiasm resulted in 336 contigs with an N50 length of 31 Mb, and the final genome size was 838.6 Mb (Table 2). Benchmarking Universal Single-Copy Orthologs (BUSCO) analysis indicated that 3572 (98.1 %) of the expected genes were found in the assembly, with 3521 (96.7 %) being single-copy and 51 (1.4 %) duplicated after searching against the actinopterygii_odb10 database (Table 2). Out of the 319 Hi-C scaffolds, 24 scaffoldings were classified as chromosome-level (Table 3). The assembled genome comprises 41.45 % repeat sequences. Gene annotation was performed using Illumina RNA-Seq and PacBio Iso-Seq data, based on repeat-masked genome sequences, resulting in 34,036 protein-coding genes (Table 4, Table 5).

**Table 1:** Sequencing strategy and statistics of raw data.

Sequencing strategy	Platform	Usage	Total reads	Total base (bp)
Short-read	Illumina NovaSeq 6000	Genome size estimation	702,143,130	106,023,612,630
Long-read	PacBio Sequel II	*De novo* genome assembly	16,422,113	29,705,891,592
Hi-C	Illumina NovaSeq 6000	Hi-C assembly	250,789,660	75,236,898,000
RNA-Seq	Illumina NovaSeq 6000	Gene annotation	87,671,954	13,238,465,054
Iso-Seq	PacBio Sequel II	Gene annotation	3654,503	4567,797,252

**Table 2:** Statistics for genome assembly.

***De novo* assembly**	
Sequencing platform	PacBio (HiFi)
Assembler	Hifiasm v0.16.1-r375
Final genome size (bp)	838,569,924
Contig numbers	336
Longest scaffold length (bp)	47,596,783
Scaffold N50 (bp)	31,873,553
GC content (%)	37.86

**Genome assembly validation**	
Complete BUSCOs (%)	3572(98.1 %)
Complete & single copy	3521(96.7 %)
Complete & duplicated	51(1.4 %)
Fragmented	18(0.5 %)
Missing	50(1.4 %)

**Table 3:** Chromosome scale of Hi-C scaffolding.

Sequencing platform	Illumina NovaSeq 6000
Software	Juicebox v2.20.00
Number of sequences	319
Scaffolds(pseudomolecule) No.	24
Total length (bp)	824,428,551 (98.31%)
Total scaffolds length	14,143,473
Minimum length	13,094
Maximum length	47,632,145
N50	33,346,874

**Table 4:** Statistics of transcriptome processing.

	RNA-Seq	Iso-Seq
Processing	Trinity assembly	Clustering with 95 % identities
Contigs number	211,667	201,849
Contigs length (bp)	211,578,101	302,217,686
Min length (bp)	189	96
Max length (bp)	40,464	11,540
Average length (bp)	1000	1497

**Table 5:** Final gene annotation.

**Final predicted genes statistics**	
Number of genes	34,036
Total length of genes (bp)	48,030,879
Smallest gene length (bp)	102
Largest gene length (bp)	92,250
Average gene length (bp)	1411
GC content (%)	50.32

**Validation by BUSCO**	
Complete BUSCOs (%)	89.4
Complete & single copy (%)	87.9
Complete & duplicated (%)	1.5
Fragmented (%)	4.3
Missing (%)	6.3

## 4 Experimental Design, Materials and Methods

### 4.1 Sample collection and DNA extraction

Individuals of *Zacco platypus* were collected from Songpa-gu, Seoul, South Korea (37°31′28.0″ N 127°5′26.0″ E). Muscle tissues were homogenized from a specimen for the extraction of high molecular weight DNA using a conventional CTAB method. The quality of the DNA was assessed using gel electrophoresis. Species identification was carried out using a primer set (LCO1490 and HCO2198) specifically targeted to amplify the mitochondrial cytochrome c oxidase I (*COI*) gene region [5].

### 4.2 Library construction, sequencing, and assembly

The genomic DNA library was prepared following the protocol of the Illumina TruSeq Nano DNA Library preparation kit (Illumina Inc., San Diego, CA, USA). The quality of the amplified libraries was verified by capillary electrophoresis (Agilent Technologies). High-throughput sequencing was performed using an Illumina NovaSeq 6000 platform following the provided protocols for 2 × 150 paired-end sequencing. Adapter sequences and low-quality reads were trimmed using Trimmomatic v0.3.9 [6], and contaminant sequences (viral, rRNA, human, or bacteria) were removed using BBDuk v38.87 from https://jgi.doe.gov/data-and-tools/bbtools.

The single-molecule real-time sequencing (SMRT) bell (SMRT Bell) library was constructed using a PacBio SMRTbell® prep kit 3.0 (Pacific Biosciences, Menlo Park, CA, USA). To verify the size of PCR enriched fragments, we check the template size distribution by running on a 2100 Bioanalyzer using a DNA 1000 chip (Agilent Technologies). The complex was loaded onto SMRT cells (Pacific Biosciences, Sequel SMRT Cell 1 M v2) and sequenced using Sequel Sequencing Kit 2.1 (Pacific Biosciences, Sequel SMRT Cell 1 M v2). For each SMRT cell, 1 × 600 min movies were captured using the Sequel sequencing platform (Pacific Biosciences) at Phyzen (Gyeonggi, South Korea). For *de novo* assembly, the Hifiasm assembler (v0.16.1-r375) was used with parameters default [7]. Statistics of *de novo* genome assembly was shown to Table 2.

### 4.3 Hi-C sequencing and chromosome scaffolding

**Fig. 1:**
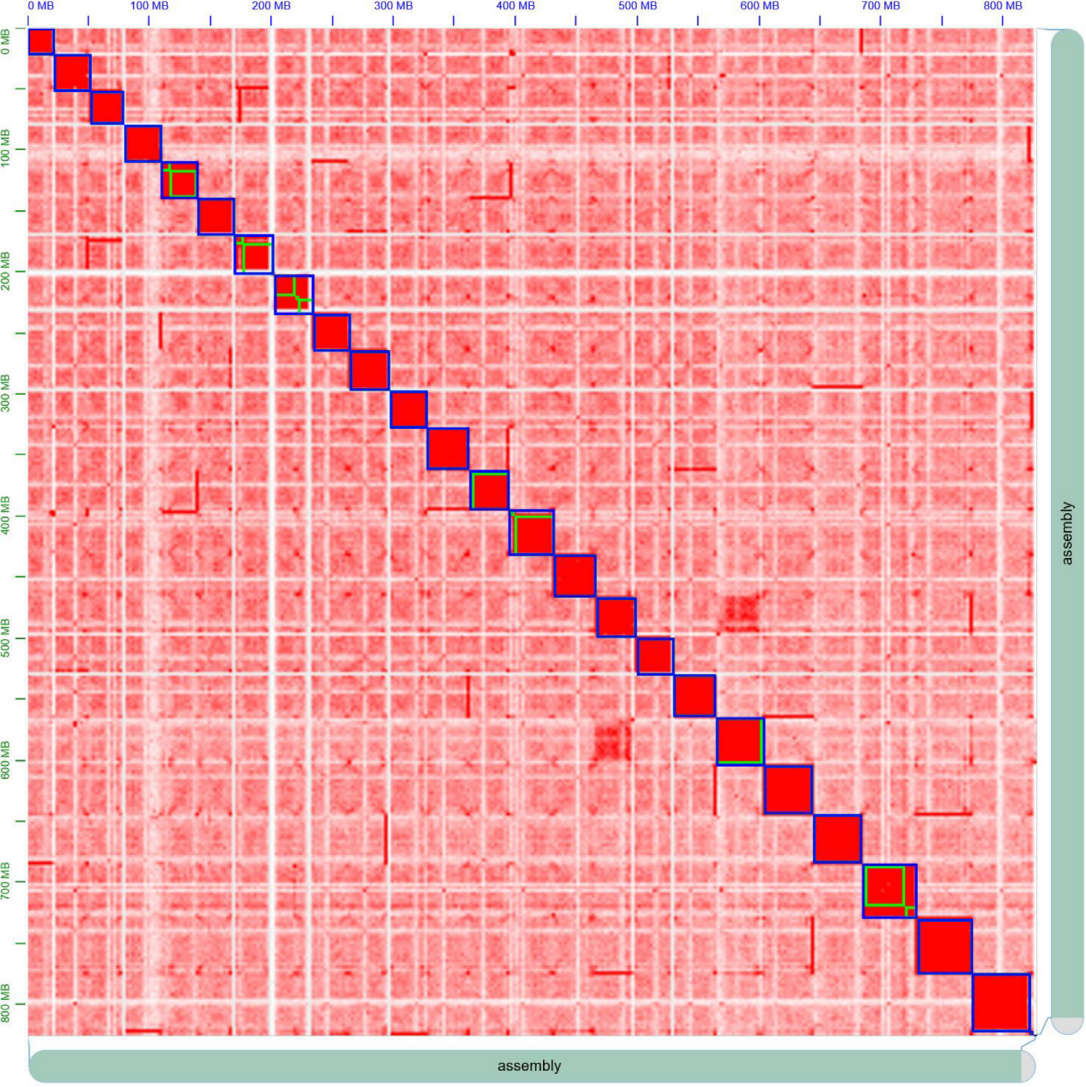
Chromosome-level genome assembly of *Zacco platypus*. Hi-C interaction heat map for *Z. platypus*. The blue boxes represent the chromosomes and green boxes indicate contig information.

Muscle tissue was frozen and ground in liquid nitrogen for the construction of the Proximo™ Hi-C library, following the instructions in the Proximo™ Hi-C kit manual (Phase Genomics, Seattle, WA, USA). Sequencing of the Hi-C library was performed on an Illumina NovaSeq 6000 platform with a 2 × 150 bp paired-end run configuration. A total of 250,789,660 Hi-C read pairs, with an aggregate length of approximately 75.24 Gb (Table 1), were aligned to the draft genome assembly using BWA [8]. Subsequently, mapping of Hi-C data was produced using the LACHESIS [9]. This Hi-C mapping was performed polishing and visualization via Juicebox v2.20.00 [10] to finalize both the genome assembly and the Hi-C contact map (Fig. 1). Final Hi-C scaffolding measured approximately 824.4 Mb with a maximum scaffold length of 47.6 Mb. Among 319 Hi-C scaffolds, we identified 24 pseudo-chromosomes in the *Z. platypus* genome, which exceeded 10 Mb in length (Table 3).

### 4.4 Completeness assessment

To evaluate the completeness of the *Z. platypus* assembly, the assembled scaffolds were subjected to BUSCO ver. 5.0 with default parameters [11], using the conservation of a core set of genes from the fish database (actinopterygii_odb10) (Table 2).

### 4.5 Repeat analysis and genome annotation

A *de novo* repeat family was identified using RepeatModeler v1.0.340, which operated with default parameters [12]. The assembled repeat library was then utilized to mask repetitive elements via RepeatMasker v4.1.2 from http://www.repeatmasker.org.

Total RNA was also extracted from the same tissues using RNeasy Mini Kit (QIAGEN Inc., Hilden, Germany). Total RNA integrity was checked using an Agilent Technologies 2100 Bioanalyzer (Bioanalyzer, Agilent Technologies, Waldbronn, Germany). Transcriptome data were obtained with Illumina paired–end sequencing (RNA-Seq; Illumina NovaSeq 6000 platform) and PacBio Sequel II (Iso-Seq; Pacific Biosciences).

The complementary DNA (cDNA) library was prepared using TruSeq Stranded mRNA Library preparation kit (Illumina) according to the manufacturer's instructions. To obtain clean data, raw reads were filtered out by trimming low-quality reads and reads containing adapters. After decontamination by BBDuk, *de novo* transcriptome assembly was performed via Trinity v2.12.0 with default option [13].

For Iso-Seq library construction, the SMRTbell library was then prepared as per the manufacturer's protocol. The pooled samples were sequenced using one SMRT cell v3 based on P6-C4 chemistry after standard full-length cDNA (1–3 kb) library preparation, and a total of two SMRT cells were sequenced on a PacBio Sequel system (Pacific Biosciences). Demultiplexing, filtering, quality control, clustering, and polishing of the Iso-Seq sequencing data were performed using SMRT Link (ver. 6.0.0). Statistics of transcriptome processing was shown to Table 4.

Gene prediction was performed using MAKER ver. 3.01.03 with default option [14]. Detailed information on the species list used for gene prediction is appended in supplementary materials (Table S1). Subsequently, filtered evidence genes (AED ≤ 0.25) were used for *ab initio* gene prediction with GeneMark-ES v4.38 [15], SNAP v2006–07–28 [16], and Augustus v3.3.2 [17]. The first gene prediction result and the *ab initio* training data set were integrated to predict the gene model, and the EvidenceModeler (EVM) was used to weight by each data. Datasets for gene prediction were prepared *de novo* transcriptome assemblies from RNA-Seq using Trinity and Iso-Seq data by clustering with 95 % identities. The polished isoforms were subjected to secondary sequence clustering by CD-HIT-EST software v4.8.1 [18]. Total number of genes was 34,036 and complete BUSCO is 89.4 % (Table 5).

## Limitations

Not applicable.

## Ethics Statement

The work meets the ethical requirements for publication in Data in Brief. Ethical approval of the study was obtained from the Incheon National University Faculty of Experimental Animals Ethics Committee (Decision No: INU-ANIM-2023-13).

## CRediT authorship contribution statement

**Sang-Eun Nam:** Writing – original draft, Data curation. **Jae-Sung Rhee:** Supervision, Methodology, Writing – review & editing, Project administration.

## Data Availability

Chromosomal-level genome assembly data from the pale chub, Zacco platypus (Original data) (NCBI)

## Appendix Supplementary materials

Image, application 1
