# Supplementary material for: Chromosomal-level genome assembly data from the pale chub, Zacco platypus (Jordan & Evermann, 1902)
Source: Data Brief. 2024 Jun 13;55:110596. doi: 10.1016/j.dib.2024.110596 (PMC11252598; doi:10.1016/j.dib.2024.110596)
Supplement: Supplementary file 1 [file mmc1.docx]

**< Supplementary Materials >**

**Chromosomal-level genome assembly data from the pale chub, Zacco platypus (Jordan & Evermann, 1902)**

Sang-Eun Nam^1^ and Jae-Sung Rhee^1,2,3,*^

*^1^ Department of Marine Science, College of Natural Sciences, Incheon National University, Incheon 22012, Republic of Korea*

*^2^ Research Institute of Basic Sciences, Incheon National University, Incheon 22012, Republic of Korea*

*^3^ Yellow Sea Research Institute, Incheon 22012, Republic of Korea*

___________________________________________________________________________

^*^Corresponding author:

Jae-Sung Rhee

Department of Marine Science, College of Natural Sciences, Incheon National University, Incheon, 22012, South Korea; Research Institute of Basic Sciences, Incheon National University, Incheon 22012, South Korea; Yellow Sea Research Institute, Incheon 22012, Republic of Korea; E-mail address: jsrhee@inu.ac.kr (J.-S. Rhee)

**Supplementary Table S1**. Relative species protein set for gene prediction.

| **Scientific name** | **Assembly** | **Assembly size (Mb)** | **Protein No.** |
| --- | --- | --- | --- |
| *Carassius auratus* | GCA_003368295.1 | 1820.64 | 96,703 |
| *Cyprinus carpio* | GCA_018340385.1 | 1680.13 | 80,686 |
| *Cyprinus carpio* | GCA_001270105.1 | 1380.1 | 50,486 |
| *Danio rerio* | GCA_000002035.4 | 1373.45 | 52,829 |
| *Labeo rohita* | GCA_022985175.1 | 1126.56 | 47,439 |
| *Labeo rohita* | GCA_004120215.1 | 1484.73 | 37,438 |
| *Onychostoma macrolepis* | GCA_012432095.1 | 886.57 | 24,754 |
| *Puntigrus tetrazona* | GCA_018831695.1 | 730.818 | 48,681 |
| *Sinocyclocheilus anshuiensis* | GCA_001515605.1 | 1632.72 | 68,474 |
| *Sinocyclocheilus grahami* | GCA_001515645.1 | 1750.29 | 67,410 |
| *Sinocyclocheilus rhinocerous* | GCA_001515625.1 | 1655.79 | 68,562 |
